# Heart rate and blood pressure in male Ts65Dn mice: a model to investigate cardiovascular responses in Down syndrome

**DOI:** 10.14814/phy2.14205

**Published:** 2019-09-08

**Authors:** Lara R. DeRuisseau, Candace N. Receno, Kevin S. Heffernan, Caitlin M. Cunningham

**Affiliations:** ^1^ Department of Biological Sciences Le Moyne College Syracuse New York; ^2^ Department of Exercise Science Syracuse University Syracuse New York; ^3^ Department of Mathematics, Statistics, and Computer Science Le Moyne College Syracuse New York

**Keywords:** Aging, arterial stiffness, autonomic nervous system, circadian, pulse wave velocity, spectral analysis

## Abstract

Down syndrome (Ds) is the most common chromosomal cause of intellectual disability that results from triplication of chromosome 21 genes. Lower blood pressure (BP) and heart rate (HR) in response to exercise and other stressors are prevalent in Ds, and are mediated by autonomic dysfunction. The Ts65Dn mouse is a model of Ds that is commonly used in preclinical studies, but has not been formally investigated for cardiovascular responses in conscious mice. Based on human studies of Ds, we hypothesized Ts65Dn would have lower BP and HR, but similar arterial stiffness. BP was quantified in conscious wild‐type (WT) and Ts65Dn. A main effect for strain was observed for all BP measures (systolic, diastolic, mean, pulse pressure), with WT higher than Ts65Dn. Pulse wave velocity was similar between WT and Ts65Dn. High‐frequency power spectra was higher in WT suggesting autonomic differences between strains. Freely moving HR was higher in WT versus Ts65Dn in both the dark and light cycles, although a main effect of circadian cycle was also present (dark> light). Similar to what is observed in humans, Ts65Dn has a lower BP which may be attributed to autonomic differences and result in preservation of arterial function with advancing age. Ts65Dn thus appears to capture the Ds cardiovascular phenotype across the lifespan. These data support further use of Ts65Dn to investigate mechanisms that may lead to altered BP and HR responses in Ds.

## Introduction

Down Syndrome (Ds) is the most common genetic cause of intellectual disability (Egan et al. 2011). Apart from cognitive deficits, individuals with Ds present with other health concerns including cardiovascular alterations (reviewed: (Versacci et al. 2018)). Congenital heart defects are observed in 40–50% of babies with Ds (Santoro et al. 2018). Low blood pressure (BP) is also reported in Ds at rest, along with reduced heart rate (HR) and/or BP responses during exercise, cold pressor and tilt tests, due to autonomic dysregulation (Fernhall and Otterstetter 2003; Iellamo et al. 2005; Bunsawat and Baynard, 2016; Hilgenkamp and Baynard 2018). Activities of daily living, including the transition from sitting to standing, require regulation of cardiovascular responses. The blunted cardiovascular response can be detrimental to individuals with Ds because it results in a reduced work capacity and quality of life (Fernhall et al. 2013).

Despite altered cardiac autonomic responsiveness, people with Ds present with preserved vascular function manifesting as no differences in arterial stiffness compared to age‐matched non‐Ds peers (Rodrigues et al. 2011). This finding is important because arterial stiffness is a recognized risk factor for future cardiovascular events (Townsend et al. 2015). Interestingly, it has been suggested that persons with Ds may be protected against atherosclerosis (Versacci et al. 2018). Thus, understanding the complex cardiovascular phenotype in Ds may be important for improving quality of life in Ds and offer insight into the pathogenesis of atherosclerotic cardiovascular disease.

Extensive BP studies have been performed on individuals with Ds demonstrating lower BP in this population (Fernhall and Otterstetter 2003; Iellamo et al. 2005; Bunsawat and Baynard 2016; Hilgenkamp and Baynard 2018). However, mechanistic studies are less common in this cohort. Some research questions about Ds would be more appropriately studied in an animal model. For instance, autonomic differences are reported in Ds, but firm conclusions about the sympathetic and parasympathetic contributions at rest are elusive since the clinical literature is conflicting (Baynard et al. 2004; Goulopoulou et al. 2006; Giagkoudaki et al. 2010; Bunsawat and Baynard 2016; Carvalho et al. 2018). The discrepancies may lie in the various control groups used (age matched, heart rate matched, people with intellectual disability without Ds, etc.) and support a rationale to also pursue HR and BP in an animal model where certain variables could be more controlled.

The Ts65Dn mouse is a model of Ds and results in triplication of the Ds critical region. It is the most cited model of Ds as it recapitulates many aspects of the Ds phenotype (reviewed: (Ruparelia et al. 2013)). Our investigation was designed to include measures that can be collected over the lifespan of a mouse. Conscious BP and freely moving HR were quantified in awake mice, while pulse wave velocity (PWV) and spectral analysis were collected under 1% isoflurane. PWV captures an important aspect of vascular aging, and subclinical atherosclerosis is predictive of MI, stroke, cognitive decline, Alzheimer's, and dementia (Townsend et al. 2015), all clinically important outcomes in the Ds population. Spectral analysis quantifies changes in autonomic function, which is a contributor to BP differences in Ds.

The overarching goal of our study was to determine if the Ts65Dn mouse displays a cardiovascular phenotype similar to people with Ds. First, we hypothesized that wild‐type (WT) mice would have higher BP compared with Ts65Dn mice at 6 and 12 months of age. A proof of concept study was extended to investigate PWV to explore if Ts65Dn has the unique vascular phenotype of Ds. We also analyzed ECG in this cohort to quantify low and high frequency HR power spectra, hypothesizing differences between WT and Ts65Dn. Lastly, to answer questions associated with cardiovascular responses across the circadian cycle, HR during the light (resting) and dark (active) cycles were compared. We hypothesized the more active periods (dark cycle) would result in potentiated differences between the groups of mice.

## Methods

### Animals

WT and Ts65Dn male mice were delivered from The Jackson Laboratory at ~2 months and aged in the facility. Mice were kept on a 12‐h light and 12‐h dark cycle, group housed in a room with only male mice, and fed ad libitum with standard rodent chow (Harlan, Teklad 2215 Rodent Diet 8640) with constant access to water. Some mice did not survive to 12 months and therefore a smaller *n* is included for this group. Additionally, not all mice habituated to the conscious experiments which influenced the n across protocols. All procedures were approved by the Le Moyne College IACUC.

### Blood pressure

Six (*n* = 14/group) and 12‐month‐old (*n* = 13 WT, *n* = 8 Ts65Dn) mice were tested for conscious systolic BP (SBP), diastolic BP (DBP), mean arterial pressure (MAP), and pulse pressure (PP; SBP‐DBP). BP was measured and analyzed as previously reported (Loeven et al. 2018) using the Kent Scientific Coda BP System (Torrington, CT). A trial was considered successful if ≥7 of the 15 inflation/deflation cycles was accepted. Previous data collected from our laboratory show similar BP values are obtained from a single trial when compared with three separate trials (Loeven et al. 2018).

### Freely moving heart rate

At 6 (*n* = 8 WT, *n* = 6 Ts65Dn) and 12 months (*n* = 11 WT, *n* = 6 Ts65Dn), HR was quantified using the MouseOx (Starr Life Sciences, Oakmont, PA). We have previously reported using this device with similar procedures in C57BL/6J (Loeven et al. 2018). Briefly, mice were tested in a range within hours 4–7 of the light and 4–7 of the dark cycle in a cylindrical testing chamber (diameter: 12 inches) with the collar in place. Typical bedding (a portion from the home cage), food and water were accessible throughout the experiment.

### Pulse wave velocity and spectral analysis

Arterial stiffness (*n* = 7/group) and HR spectral analysis (*n* = 8/group) were analyzed under light isoflurane anesthesia in mice at 9 months. After induction of isoflurane, mice were placed on a heating pad in the supine position and fur was removed from the skin surrounding the left groin area. A 5‐lead electrocardiogram (ECG) with needle electrodes was applied to the mouse and connected to an iWorx animal physiology signal conditioner/amplifier (Dover, NH). A calibrated SPT‐310 Millar (Millar, Inc., Houston, TX) tonometer probe fitted to a TC‐510 passive pressure control unit was integrated into another iWorx channel. This setup allowed for simultaneous collection of ECG and pulse waves. The tonometer was held in place with a micromanipulator (Narishige, Amityville, NY) and small adjustments were made when required to locate the pulse. After proper placement of the ECG electrodes and confirmed pulse wave with the tonometer, isoflurane was lowered to 1% for 11 min. We have previously reported no BP differences with 1% isoflurane, and breathing recovery by 11 min (Loeven et al. 2018). Breathing frequency and HR were monitored for the duration of the experiment. PWV was analyzed at the 11th minute of data collection. For PWV analysis, we followed a modified method reported by Leloup and colleagues (2014). The time from the R peak to the foot of the pulse wave was measured for 15 sec of data. The distance from the heart to the femoral artery (exact location of tonometer) was measured in cm. To calculate PWV, the distance travelled was divided by the average time measured (ECG to pulse wave; sec) and then normalized to the MAP for each mouse.

For heart rate spectral analysis, 1 min of stable ECG recordings at 11 min were exported to Kubios Heart Rate Variability software (version 3.2). We followed the method to evaluate ~60 sec of stable heart rates (Billman, 2013; Jaimes et al. 2017) when mice have consistent behavior using the low (LF: 0.15–0.6 Hz) and high frequency (HF: 2.5–5.0 Hz) power (msec^2^) spectra. This method was first described by Baudrie et al. (2007) using HR similar to the values we report.

### Statistical analysis

For the 6‐ and 12‐month BP comparisons, a mixed model with R was utilized (Measure*Age*Type as fixed effects, with Mouse as a random effect, with Age nested within Mouse). The three measures (DBP, SBP, MAP) were run simultaneously to account for the covariate structure, but we were also interested in examining parameters separately. We did this by examining combinations of coefficients. Pulse pressure was analyzed separately using the same mixed model. To detect possible differences for PWV, a Student's *t*‐test compared WT and Ts65Dn. Spectral analysis data was evaluated using multivariate ANOVA. HR was analyzed in the 6‐ and 12‐month‐old mice using a three‐way ANOVA with a between and within subjects mixed model. Data are presented as mean ± standard deviation.

## Results

### Blood pressure

**Figure 1:**
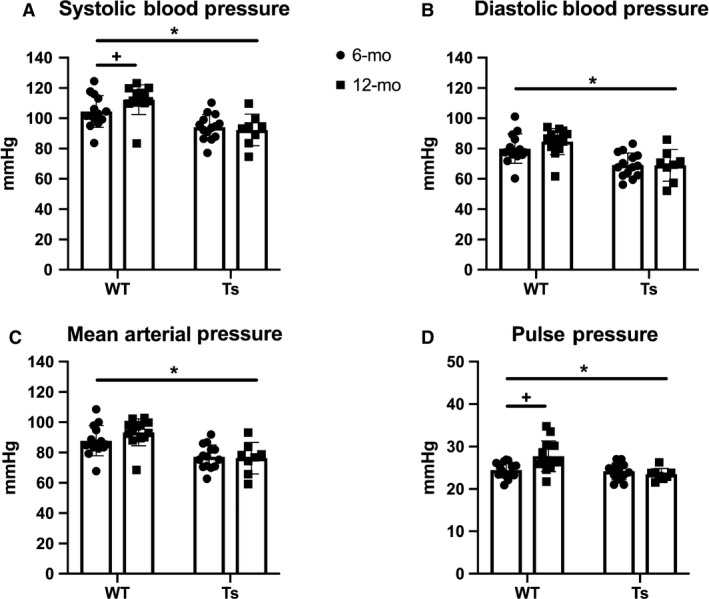
Blood pressure (BP) at 6 and 12 months is higher in wild‐type (WT) versus Ts65Dn (Ts) mice. Conscious (A) systolic BP (SBP; mmHg), (B) diastolic BP (DBP; mmHg), (C) mean arterial pressure (MAP; mmHg) and (D) pulse pressure (PP; SBP‐DBP; mmHg) were collected using a Coda BP system in WT and Ts mice. WT mice had higher values compared with Ts (*P < 0.05). Twelve‐month‐old WT mice had higher SBP and PP compared to 6‐month olds (^+^ *P* < 0.05). Data were analyzed using a mixed model and presented as mean ± SD.

BP quantification in WT and Ts65Dn at 6‐ and 12‐month‐old mice revealed a main effect of strain for diastolic (DBP; *P* < 0.001), systolic (SBP; *P* < 0.001), mean arterial pressure (MAP; *P* < 0.001), and pulse pressure (PP: *P* = 0.0027), and an additional strain by age interaction for SBP (*P* = 0.0147) and pulse pressure (PP; *P* = 0.0142). Ts65Dn were lower compared with WT for all of these measures (Fig. 1). A post hoc paired *t*‐test on the mice in both age groups confirm that as WT mice age, their SBP and PP become elevated (SBP: *P* < 0.001; PP: *P* = 0.013).

### Pulse wave velocity

**Figure 2:**
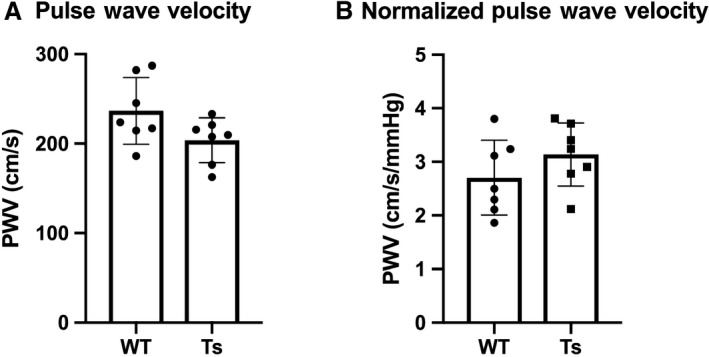
Pulse wave velocity (PWV) is not different in wild‐type (WT) and Ts65Dn (Ts) mice. Data were collected and analyzed for (A) non‐normalized: cm/sec, and (B) normalized: cm/sec/mmHg PWV from lightly anesthetized (1% isoflurane) mice, and were not statistically different between groups (*P*> 0.05). Data points ≥3 SD from mean were considered outliers. One outlier (Ts mouse) excluded from PWV analysis. Data were analyzed using t‐tests and presented as mean ± SD.

Non‐normalized PWV (cm/sec) and normalized (PWV/MAP; cm/sec/mmHg) uncovered no differences between WT and Ts65Dn (Fig. 2, *P* > 0.05).

### Spectral analysis

Analysis of HF power (msec^2^) uncovered differences between groups, with WT higher than Ts65Dn (*P* = 0.021; Table 1). We performed an additional analysis using the recommendations for awake and moving mice exhibiting different behaviors (Thireau et al. 2008), and also observed differences for HF (*P* = 0.048). However, the latter analysis is likely not ideal based on our HR range and uniform behavior observed during 1% isoflurane anesthesia. No differences for LF (msec^2^) or LF/HF ratio were observed (*P* > 0.05) with either analysis.

**Table 1:** Heart rate spectral analysis

	RR interval (msec)	HR (bpm)	HF (msec^2^)	LF (msec^2^)	HF/LF ratio
WT	127.14 ± 17.27	480 ± 70	1.89 ± 1.45^*^	0.96 ± 1.21	0.59 ± 0.72
Ts	120.50 ± 30.19	483 ± 81	0.51 ± 0.34	0.70 ± 0.75	1.49 ± 1.81

Spectral analysis of 9‐month‐old wild‐type (WT) and Ts65Dn (Ts) mice (*n* = 8 WT; *n* = 8 Ts). Data were collected from lightly anesthetized (1% isoflurane) mice. High frequency (HF) power was significantly higher in WT versus Ts mice (*P < 0.05). Low frequency (LF) and HF/LF ratio were not significantly different between groups (*P*> 0.05). Values are mean ± SD.

### Heart rate

**Figure 3:**
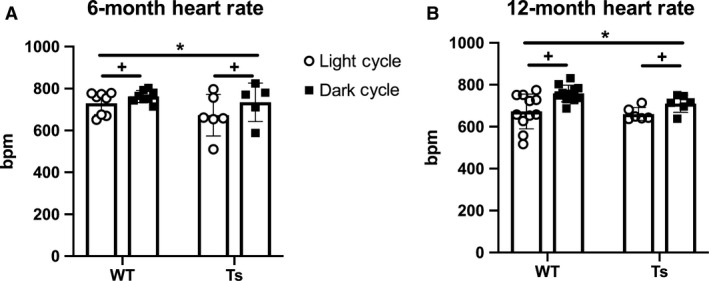
Freely moving heart rate (HR) is higher in wild‐type (WT) versus Ts65Dn (Ts) mice. Collars were placed on (A) Six and (B) Twelve‐month‐old mice for continuous monitoring of HR during a range within the light cycle (hours 4–7) and dark cycle (hours 4–7). Data points ≥3 SD from mean were considered outliers. One Ts mouse (6‐month) excluded from dark cycle analysis. Higher HR was observed in WT compared with Ts mice (*P < 0.05) and dark cycle HR was elevated versus the light cycle HR (^+^ *P* < 0.05). Data were analyzed using three‐way ANOVA and presented as mean ± SD.

Analysis of freely moving HR at 6 and 12 months of age resulted in main effects of both light‐dark cycle and mouse strain (Fig. 3). We observed higher HR in WT and Ts65Dn mice during the dark cycle compared to the light cycle (*P* = 0.003). WT mice displayed an overall higher HR versus Ts65Dn (*P* = 0.048) with no effect of age (*P* = 0.150). No interaction was detected, so the response in the light or dark cycles does not appear to depend on the strain. This finding is in contrast to our hypothesis that Ts65Dn would have an attenuated HR during the dark cycle. The main effect of strain does support our hypothesis that Ts65Dn would have a lower HR overall compared to WT.

## Discussion

This investigation revealed the cardiovascular phenotype of Ts65Dn compared with their WT controls. BP quantification resulted in higher values in WT versus Ts65Dn that was potentiated as mice aged. While distinct BP differences emerged between the groups, PWV was similar. The high frequency HR spectra were higher in WT, suggesting different autonomic regulation in this group. We observed higher freely moving HR in WT versus Ts65Dn. Additionally, HRs in the dark were higher than those in the light cycle. Taken altogether, these data demonstrate Ts65Dn is an appropriate model to continue investigating BP and HR regulation in Ds.

Lower BP in Ts65Dn at 6 and 12 months is in line with reports demonstrating lower BP across the lifespan in Ds (Heffernan et al. 2005; Bunsawat and Baynard, 2016). People with Ds have been reported to experience lower BP at rest, and with exercise, due to autonomic dysregulation (Heffernan et al. 2005; Bunsawat and Baynard, 2016). In older individuals without intellectual disability, lower BP is associated with reduced cognitive functioning (Nilsson et al. 2007; Axelsson et al. 2008) and it is unknown if lower BP influences dementia in Ds aging. Since the life expectancy is increased for Ds (Covelli et al. 2016), and many individuals with Ds will have some form of dementia (Hithersay et al. 2019), understanding how BP is regulated across the lifespan will be important for this population. These data demonstrate Ts65Dn are a useful and important tool to understand more mechanistic underpinnings of BP regulation in Ds.

To begin unraveling a mechanistic component to BP, we collected additional cardiovascular data by quantifying PWV in WT and Ts65Dn as a measure of arterial stiffness. We chose 9 months for these experiments, using the mid‐point between the other ages since these measures were included as a proof of concept study. These data were not different across groups, similar to findings in Ds. Although Ds is associated with dyslipidemia (Pueschel et al. 1992; Adelekan et al. 2012), older people with Ds are protected from atherosclerosis and heart disease (Versacci et al. 2018), and have normal arterial stiffness (once normalized to the lower BP (Rodrigues et al. 2011; Parra et al. 2017)). Therefore, our data show that Ts65Dn could be useful to study atherogenesis and novel factors responsible for cardioprotection in Ds.

Autonomic dysregulation is a known contributor to lower BP in Ds, particularly during times of increased demand (Fernhall and Otterstetter 2003; Iellamo et al. 2005; Bunsawat and Baynard 2016; Hilgenkamp and Baynard 2018). However, resting sympathetic and parasympathetic control is not standardized across studies although some reports suggest higher parasympathetic tone in controls versus Ds (Goulopoulou et al. 2006; Giagkoudaki et al. 2010; Carvalho et al. 2018). Our proof of concept data suggest that autonomic function measured via HR high frequency power spectra is different between WT and Ts65Dn under 1% isoflurane anesthesia. We have recently reported light anesthesia results in similar blood pressure, P_O2_ and P_CO2_ at 11 min with 1% isoflurane (Loeven et al. 2018). Therefore, we tested mice at this same timepoint. HR is still reduced with 1% isoflurane anesthesia, resulting in similar HR for WT and Ts65Dn. We did not consider the lower HR inherently negative since differences in HR can influence HRV analysis (Monfredi et al. 2014; Gasior et al. 2016). The light anesthesia resulted in similar HRs across groups for this initial analysis. Our data show higher high frequency power spectra in WT versus Ts65Dn, suggesting greater parasympathetic tone in WT. Increased parasympathetic control during 1% isoflurane in WT would result in a greater ability and range to increase HR with parasympathetic withdrawal. This possibility is in line with the data we report showing higher HR in awake WT mice versus Ts65Dn.

We agree that future studies of HRV could include other physiological states in awake mice, but the differences in HR would need to be accounted for in the analyses (Sacha and Pluta 2008). While multiple comparisons have been reported using HRV in people with Ds (Baynard et al. 2004; Goulopoulou et al. 2006; Giagkoudaki et al. 2010; Bunsawat and Baynard 2016; Carvalho et al. 2018), there is not a widespread acceptance of baseline autonomic dysfunction in this population. Therefore, it is not possible to directly compare the mouse data to the clinical literature. The experiments we conducted were intended to initially identify possible autonomic differences between WT and Ts65Dn in one physiological state. Future studies could include conscious, anesthetized and sleep states as well as autonomic blockade to determine the sympathetic and parasympathetic contributions to lower HR and BP in Ts65Dn.

The lower BP and HR during activities of daily living in Ds are associated with a lower quality of life and is a clinical concern for this cohort. However, a life of lower BP and HR in Ds at rest and in response to perturbation may help preserve vascular function (lower distending pressure and thus chronic cyclic stress/strain). These reports beg the question: is the cardiovascular phenotype in Ds protective, or pathological, or a combination of the two? The answers are likely to be multifactorial and we suggest the Ts65Dn mouse is a way to answer additional research questions in this biomedical research area.

In order to determine if Ts65Dn mice exhibit lower HR, we studied freely moving mice during the light and dark cycles. These data suggest that Ts65Dn may have lower hemodynamic strain (HRxBP) across the circadian cycle. Fragmented ECG has been reported in anesthetized Ts65Dn (Raveau et al. 2012) suggesting that electrical activity could be contributing to altered HR in Ts65Dn. Ventricular abnormalities are also observed in Ts65Dn neonates (Williams et al. 2008) that perish shortly after birth and it is plausible that surviving Ts65Dn have functional and/or anatomical differences. Disparities in the pumping action of the heart and subsequent blood flow could be influencing both HR and BP.

The studies we describe here include relatively non‐invasive techniques. The reasons for this experimental design were multifaceted: (1) These measures allow for collection across multiple ages in an expensive mouse strain, and (2) Ts65Dn are more fragile with respect to surgical procedures precluding use of other measures (e.g., telemetry) in this initial study. We have previously reported (Loeven et al. 2018) tail BP to be repeatable across three testing days with a low standard deviation and used the technique in these studies with assurance. However, it is possible that WT and Ts65Dn respond to restraint differently. While tail BP methods correspond to central pressure in C57BL/6J mice (Wilde et al. 2017), it is possible that Ts65Dn have a unique response to this technique. Perhaps Ts65Dn tail BP more closely resembles their response (or lack thereof) to a stressful situation. Nonetheless, the data demonstrate a lower BP for Ts65Dn in this scenario. Future investigations could focus on radiotelemetry to identify BP regulation in unrestrained Ts65Dn and across the circadian cycle. To study mice that were freely moving, we added HR with a tethered collar. Taken altogether, the relatively non‐invasive techniques have benefits that suited our studies. Future investigations could certainly include other surgeries (terminal or recovery) to understand mechanistic changes in Ts65Dn, and as a correlate, Ds.

These initial integrative physiological experiments now open the door for more cardiovascular studies in Ts65Dn. We report a HR and BP profile in Ts65Dn that appears to model findings in people with Ds. A slightly higher BP profile, but still within a normal range, is a predictor of Myamoya syndrome and stroke in Ds (Santoro et al. 2018). Therefore, the lower resting BP in Ds may be normal for this population and understanding situations when this baseline value might change is vital. The inability to increase BP and HR in times of physiological demand is a feature that affects the daily living of people with Ds and influences their ability to be active (Shields et al. 2018). Future studies to explore these questions will be critical for this population, and should include Ts65Dn females since males and females have different heart abnormalities in Ds (Santoro et al. 2018) and it is unknown how these differences at birth may alter future cardiovascular physiology. Finding ways to explore more mechanistic questions about Ds will be critical to understanding cardiovascular regulation in this population. The Ts65Dn mouse model may offer these opportunities.

## Conflict of Interest

None of the authors has any conflict of interest.
